# *De novo* transcriptome sequencing and gene expression analysis reveal potential mechanisms of seed abortion in dove tree (*Davidia involucrata* Baill.)

**DOI:** 10.1186/s12870-016-0772-x

**Published:** 2016-04-12

**Authors:** Meng Li, Xujie Dong, Jiqing Peng, Wen Xu, Rui Ren, Jane Liu, Fuxiang Cao, Zhiming Liu

**Affiliations:** Key Laboratory of Cultivation and Protection for Non-wood Forest Trees, Ministry of Education, College of Life Science and Technology; Central South University of Forestry and Technology, Changsha, People’s Republic of China; Department of Biology, Eastern New Mexico University, Portales, NM 88130 USA

**Keywords:** Transcriptome, Adversity stress, Phytohormone, Seed abortion, Integument, Dove tree (*Davidia involucrata* Baill.)

## Abstract

**Background:**

Dove tree (*Davidia involucrata* Baill.) is a rare and endangered species. Natural reproduction of dove tree is extremely difficult due to its low fecundity. Serious seed abortion is one of the key factors restraining its sexual reproduction. Understanding the inducements of seed abortion is critical for addressing the issue of offspring production and the survivability of such an endangered species. However, studies on the molecular mechanism of seed abortion in woody plants are lacking, and the dearth of genomic resources for dove tree restricts further research.

**Results:**

In this study, using the Illumina platform, we performed *de novo* transcriptome sequencing of the fruit and seed in dove tree. A total of 149,099 transcripts were isolated and then assembled into 72,885 unigenes. Subsequently, differentially expressed genes (DEGs) between normal and abortive seeds were screened. Genes involved in response to stress, hormone signal transduction, programmed cell death, lignin biosynthesis, and secondary cell wall biogenesis showed significant different expression levels between normal and abortive seeds.

**Conclusion:**

Combined results indicated that the abortive seeds were under the adversity stress, which should be controlled by the maternal plant. Maternally controlled development of integument is assumed to be a critical process for abortion regulation. MYB and WRKY transcription factors, receptor kinase and laccase are considered to be important regulators in seed abortion. Moreover, mass sequence data facilitated further molecular research on this unique species.

**Electronic supplementary material:**

The online version of this article (doi:10.1186/s12870-016-0772-x) contains supplementary material, which is available to authorized users.

## Background

*Davidia involucrata* Baill., also known as dove tree or handkerchief tree, is a relic species of the Tertiary [1]. *Davidia* was a dominant part of the flora at many sites in the Paleocene of North America. However, it is demic to China today [1, 2]. It is considered by most researchers to be the sole member of the genus *Davidia* of the family *Davidiaceae* [3]. The most special characteristics of dove tree are its head inflorescences and intriguing pair of white bracts. *Davidia* is also an endangered species that has been listed as a first-grade nationally protected plant of China [2]. Currently, the distribution of natural *Davidia* population is rare and scattered, mainly due to its rigorous ecotope demand and low fecundity. In China, distribution areas of natural dove tree population are continuously decreasing, and most natural populations present the “Inverted Pyramid” structure, which indicates population depression [4]. For dove tree resources conservation, introduction and artificial breeding techniques of *Davidia* have been studied in China since 1979 [5]. However, studies did not progress smoothly as *Davidia* sexual reproduction was seriously restricted by the extremely long dormancy periods and high abortion ratio of its seeds [5]. Generally, only 1–3 well-developed seeds could be found in a *Davidia* fruit. Our observation found the manner of seed abortion in *Davidia* was independent with temperature, precipitation, biennial cycle and genotype. Moreover, seed abortion occurred in other endangered tree species such as *Caryocar brasiliense* [6], *Magnolia denudate* [7] and *Liriodendron chinense* [8], implying conserved mechanisms of seed abortion existed within these rare species.

Flower, fruit and seed abortion is pervasive in the plant kingdom. Many plant species, especially perennials, produce far more flowers than fruits and more ovules than seeds [9]. The low seed to ovule and fruit to flower ratios cause poor fecundity in some long-living tree species [10]. Evolutionary hypotheses propose that this “surplus of flowers or ovules” is a bet-hedging strategy that accounts for variable and unpredictable environments [11]. Diverse explanations have been proposed to interpret the mechanism underlying this phenomenon, including resource limitation [12, 13], pollen deficiency [14, 15], sibling rivalry [16] and genetic load [10, 17, 18]. Seed abortion could occur at different developmental stages of the embryo due to genotype, low vigor, inferior position or pathogen infection [19]. Abortion is considered to be a potentially beneficial mechanism that increases progeny quality [11]. Recent reports suggest that seed abortion is a complex plant behavior triggered by internal and external conditional cues [20]. However, for endangered species, such abortion mechanisms seriously limit proliferation, cultivation and conservation.

Despite numerous studies on seed abortion, most are focused on the physiological and morphological rather than molecular level. This is partly due to the fact that species with serious seed abortion are usually non-model plants, leading to a lack of genomic data. Recently research has focused on the genes and proteins involved in seed abortion in longan [21], peanut [22], chrysanthemum [23] and hazelnut [24] using transcriptome and proteome analysis.

To reveal the molecular events occurring in abortive seeds of *Davidia*, we used the Illumina platform and *de novo* sequenced the transcriptome to establish the first unigene library of fruit and seed of *Davidia*. Moreover, we identified the differentially expressed genes (DEGs) between normal and abortive seeds. Genes involved in cell proliferation, DNA replication, nutrient reservoir activity, and starch and sucrose metabolism were found to have significantly higher expression in normal seeds. In contrast, genes involved in response to stress, oxidoreductase activity, secondary metabolites biosynthesis and programmed cell death were found to be uniformly up-regulated in abortive seeds. DEGs encoding transcription factors, receptor kinase, proteinase and laccase were presumed to be critical regulators in seed abortion. These findings will bring valuable insight to the molecular regulatory mechanism of seed abortion in woody perennials.

## Results

### Seed abortion in *Davidia*

**Fig. 1:**
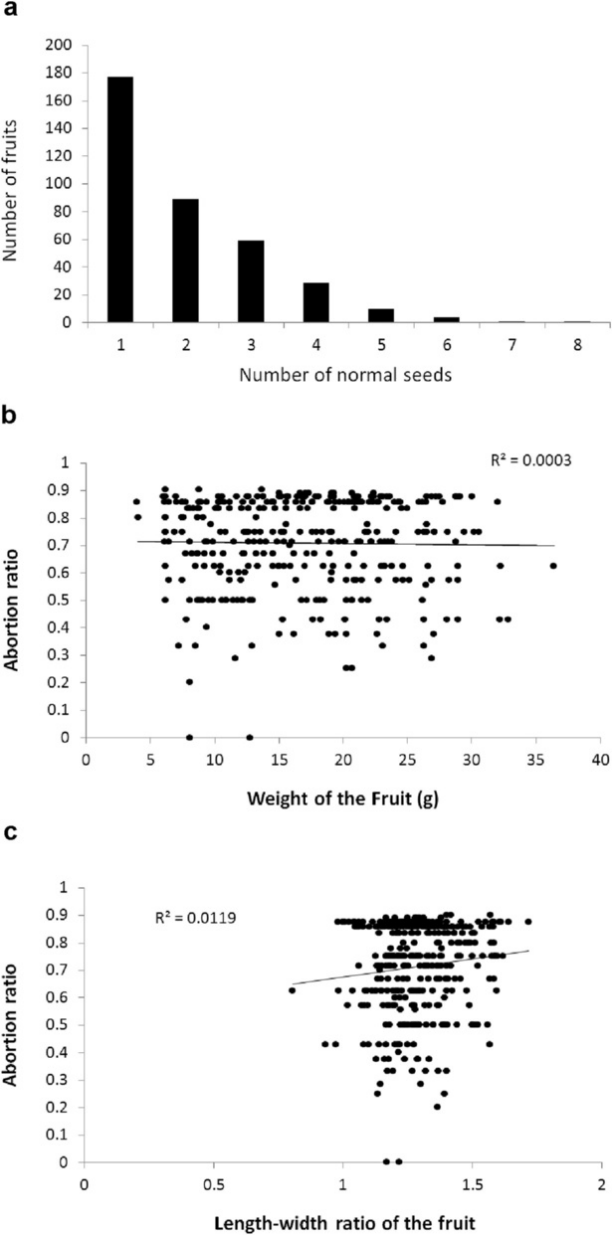
Seed abortion in *Davidia*. **a** Distribution of the numbers of normal seed in Davidia fruits; **b** Correlation between fruit weight and abortion ratio of seed; **c** Correlation between length-width ratio of fruit and abortion ratio of seed

**Fig. 2:**
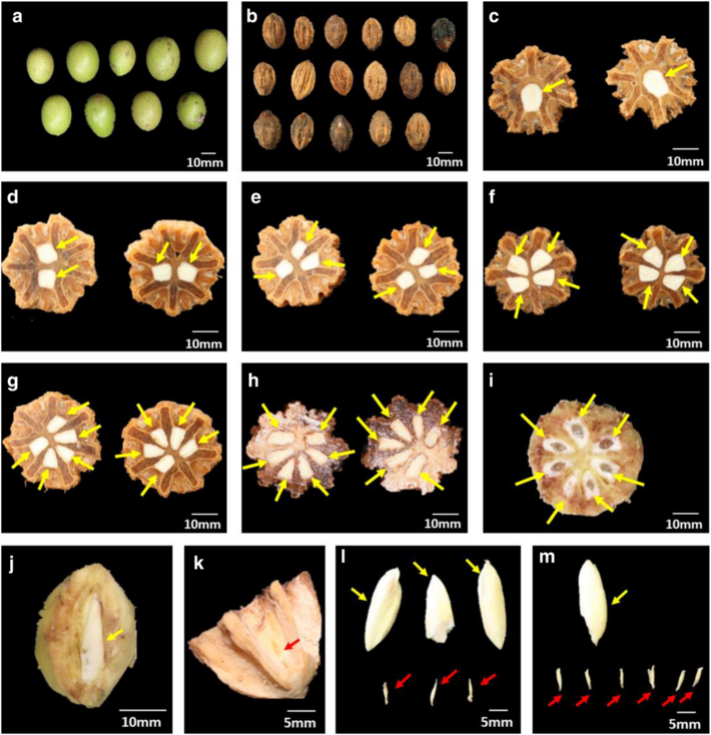
Fruits, normal seeds and abortive seeds of *Davidia.* **a** The intact fruits; **b** Fruits with sarcocarp removed; **c**-**i** Crosscutting sections of the kernels. The fruits contain 1 to 7 normal seeds are displayed in turn; **j** A normal seed in fruit; **k** An abortive seed in fruit; **l** Normal and abortive seeds collected from identical fruit. The fruit has 3 normal seeds and 3 abortive seeds; **m** Normal and abortive seeds collected from identical fruit. The fruit has 1 normal seeds and 6 abortive seeds. Normal and abortive seeds are represented by yellow and red arrows, respectively

In order to investigate seed abortion situation in *Davidia*, we collected approximately 400 fruits from more than ten individual trees and recorded the numbers of normal and abortive seeds in them. The fruit of *Davidia* has an 8-carpel structure (sometimes 1–2 carpels degenerated from pistil development). In most fruits, the number of normal seeds was 1–3. As such, more than half of the seeds were aborted (Fig. 1a). The correlation between fruit weight and abortion ratio, and length-width ratio of fruit and abortion ratio were statistically analyzed, respectively. The results showed no significant correlation between either of them, indicating that seed abortion occurred at the early developmental stage and was independent to fruit development (Fig. 1b and c). We found fruits with normal seed numbers ranging from 1–8, indicating that all ovules had potency to develop well (Fig. 2c-i). Unlike some legume plants, the distances between the stigma and each ovule were approximately equivalent in *Davidia* so all ovules had equal opportunities for nutrient uptake. Position effect, a key cause of seed abortion in some legume plants, could be eliminated in *Davidia*. Moreover, the abortion was observed to occur in either consecutive or interval carpels, implying no obvious competition among siblings (Fig. 2d-h). For appearance, the well-developed seeds were spindle-shaped, white in color and rich in fat while the abortive seeds were noticeably smaller and more shriveled, with seed coats that were tan in color (Fig. 2j-m).

**Fig. 3:**
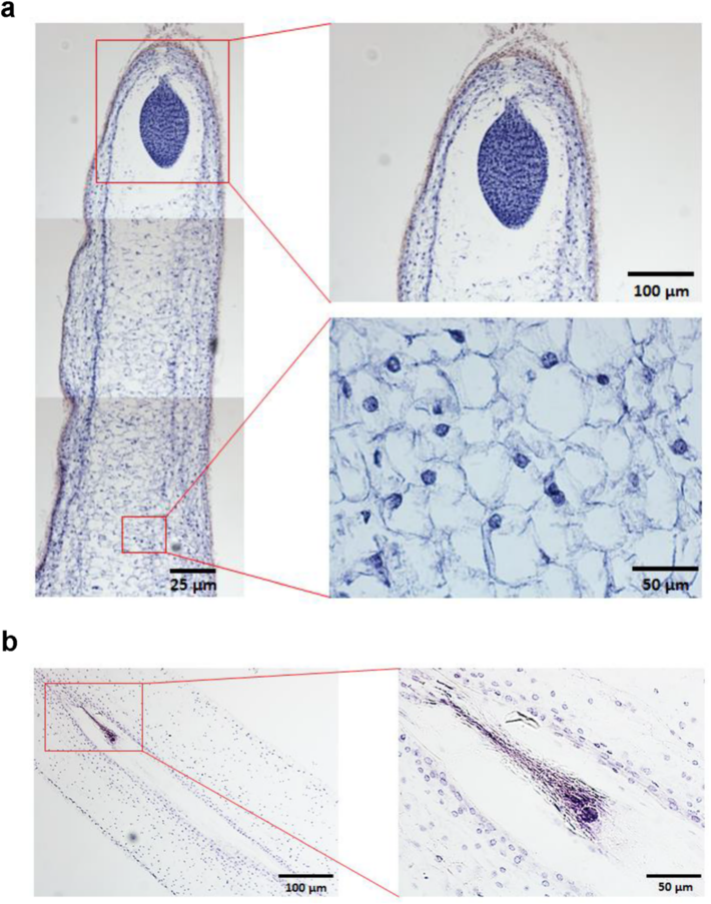
Microstructure of normal and abortive seeds. **a** The well-developed embryo and endosperm in normal seed. **b** The empty embryo sac and degenerative embryo in abortive seed

We investigated the microstructure differences between normal and abortive seeds by microscopic observation. Paraffin sections demonstrated that the embryo sac in a normal seed was stacked well and the embryo was intact. On the contrary, in an abortive seed from the same fruit, the embryo sac was empty and flat, and the egg apparatus had been totally degenerated, indicating that the seed abortion occurred at the early stage of embryo development (Fig. 3).

### Overview of the *Davidia* transcriptome

One fruit sample, three normal seed samples and three abortive seed samples of *Davidia* were used to build a mixed library for high-throughput sequencing. RNA quality of each sample, represented by RNA integrity number (RIN), was 9.6 (Di-1 N), 9.1 (Di-1A), 9.5 (Di-2 N), 9.0 (Di-2A), 9.1 (Di-3 N), 9.1 (Di-3A) and 10.0 (Di-F), respectively. RNAs of different samples were mixed in equal quantities to construct the cDNA library. In total, the library produced 6,472,538,761 (6.47G) raw data with 89.2 % Q30 bases (percentage of sequences with sequencing error rates <0.1 %) by Illumina HiSeq 2500.

Using the Trinity *de novo* assembly program, short-read sequences were assembled into 149,099 transcripts with a mean length of 1,056.57 bp. The sequencing raw data was deposited to the NCBI Short Reads Archive (SRA) with the accession number SRP058736. The transcripts were then subjected to cluster and assembly analysis. Finally, we harvested a total of 72,885 unigenes with N50 length of 1150 and an average length of 656.61 bp (Additional file 1). An overview of the assembly contigs and unigenes is shown in Table 1.

**Table 1:** Summary of Illumina transcriptome assembly for *Davidia*

Length range	Contig	Transcript	Unigene
200–300	3,948,088(98.63 %)	35,712(23.95 %)	29,975(41.13 %)
300–500	25,956(0.65 %)	26,996(18.11 %)	18,692(25.65 %)
500–1000	14,748(0.37 %)	28,175(18.90 %)	11,244(15.43 %)
1000–2000	9,357(0.23 %)	35,663(23.92 %)	8,289(11.37 %)
2000+	4,603(0.11 %)	22,553(15.13 %)	4,685(6.43 %)
Total Number	4,002,752	149,099	72,885
Total Length	218,539,083	157,533,456	47,856,850
N50 Length	49	1,766	1,150
Mean Length	54.60	1,056.57	656.61

**Fig. 4:**
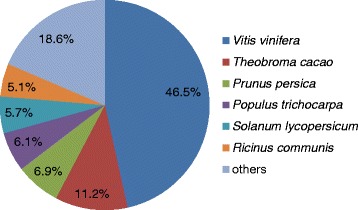
Species distribution of the BLASTX results of *Davidia* transcriptome. The numbers in the pies indicate the percentage of unique reads in each category

All the 73,885 assembled unigenes were searched against the Nr, Swiss-Prot, GO, COG and KEGG databases using the BLAST algorithm (E-value < 1E^−5^) (Table 2). Totally, 33,725 (45.6 %) unigenes were annotated (Additional file 2). Nr database queries revealed that a high percentage of *Davidia* sequences closely matched the sequences of *Vitis vinifera* (46.5 %), *Theobroma cacao* (11.2 %), *Prunus persica* (6.9 %), *Populus trichocarpa* (6.1 %), *Solanum lycopersicum* (5.7 %) and *Ricinus communis* (5.1 %) (Fig. 4). To ensure the accuracy of the annotation, the assembled unigenes were searched against the genomic database of *Arabidopsis thaliana*, *Vitis vinifera*, *Theobroma cacao*, *Populus trichocarpa*, *Eucalyptus grandis* and another relic species, *Amborella trichopoda*. 34.2 % - 39.9 % of total *Davidia* unigenes were annotated to the genomic data of these species (Additional file 3).

**Table 2:** Summary for the annotation of unigenes of Davidia

Annotated databases	Unigene	≥300 nt	≥1000 nt
COG	8,375	7,605	5,268
GO	24,834	19,635	10,157
KEGG	6,257	5,162	2,980
Swiss-Prot	21,010	17,024	8,925
Nr	33,562	25,908	12,207
All	33,725	25,983	12,210

Among annotated unigenes, 24,834 unigenes were matched in the GO database and classified into 3 functional categories: molecular function (13,386, 53.9 %), biological process (4918, 19.8 %) and cellular component (6530, 26.3 %). “Binding”, “catalytic activity” and “transporter activity” were the largest GO terms of molecular function. “Metabolic process”, “cellular process” and “response to stimulus” were the largest GO terms of biological process. And “cell part”, “cell” and “organelle” were the largest GO terms of cellular component. KEGG pathway analysis showed 6257 unigenes were matched in the database and assigned to 116 KEGG pathways. The pathways containing the largest number of unigenes include “ribosome”, “plant hormone signal transduction”, “spliceosome”, “protein processing in endoplasmic reticulum”, “RNA transport”, “oxidative phosphorylation”, “purine metabolism”, “glycolysis/gluconeogenesis”, “starch and sucrose metabolism” and “plant-pathogen interaction”.

### DEGs between normal and abortive seeds

A total of 61.27 M reads, including 3.12 G of raw data, were produced by RNA-seq. The high quality reads were aligned to the established *Davidia* unigene library and the proportions of mapped reads ranged from 74.3 % to 77.9 % (Table 3). The RPKM values of all genes were used to analyze the correlation between each of the two samples. The correlation coefficients between normal seed samples were higher than 0.82 (an exception is Di-2 N vs. Di-3 N, 0.78), and the correlation coefficients between abortive seed samples were higher than 0.94, indicating the slight variability among the biological replicates. The correlation coefficient between each normal seed sample and abortive seed sample was less than 0.10, indicating a significant expression difference (Additional file 4).

**Table 3:** Summary for the alignment of reads to unigene library

Sample	Total reads	Mapped reads	Unique mapped reads	Multiple mapped reads
Di-1N	9,617,978	7,491,843(77.89 %)	6,835,644(71.07 %)	656,199(6.82 %)
Di-1A	9,958,796	7,533,463(75.65 %)	6,759,052(67.87 %)	774,411(7.78 %)
Di-2N	10,659,112	8,156,133(76.52 %)	7,443,600(69.83 %)	712,533(6.68 %)
Di-2A	11,102,640	8,534,699(76.87 %)	7,595,828(68.41 %)	938,871(8.46)
Di-3N	9,762,626	7,249,252(74.26 %)	6,627,147(67.88)	622,105(6.37 %)
Di-3A	10,171,988	7,702,621(75.72 %)	6,919,954(68.03 %)	782,667(7.69 %)

**Fig. 5:**
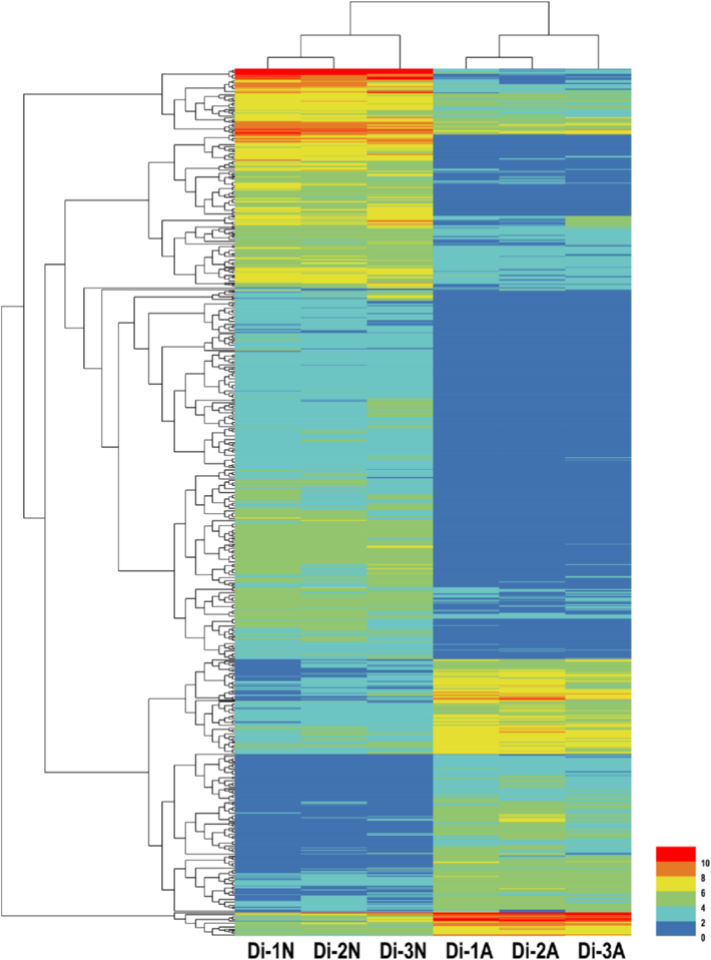
Expression pattern of DEGs in *Davidia* seeds. Hierarchical clustering analysis of DEGs between normal and abortive seeds based on expression data

**Fig. 6:**
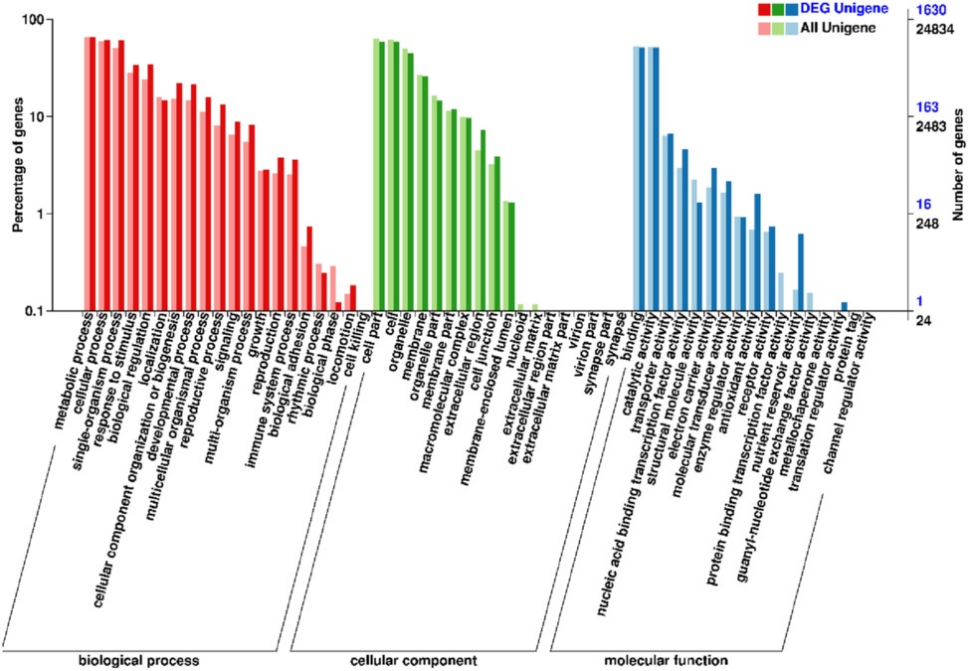
Gene Ontology classification of DEGs between normal and aborted seeds. Unigenes were annotated in three categories: cellular components, molecular functions, and biological processes. Numbers in black represent the numbers of all unigenes in GO terms, and numbers in blue represent the numers of DEGs in GO terms. DEGs were significantly enriched in the terms such as “biological adhesion”, “extracellular region”, “antioxidant activity” and “nutrient reservoir activity”

**Fig. 7:**
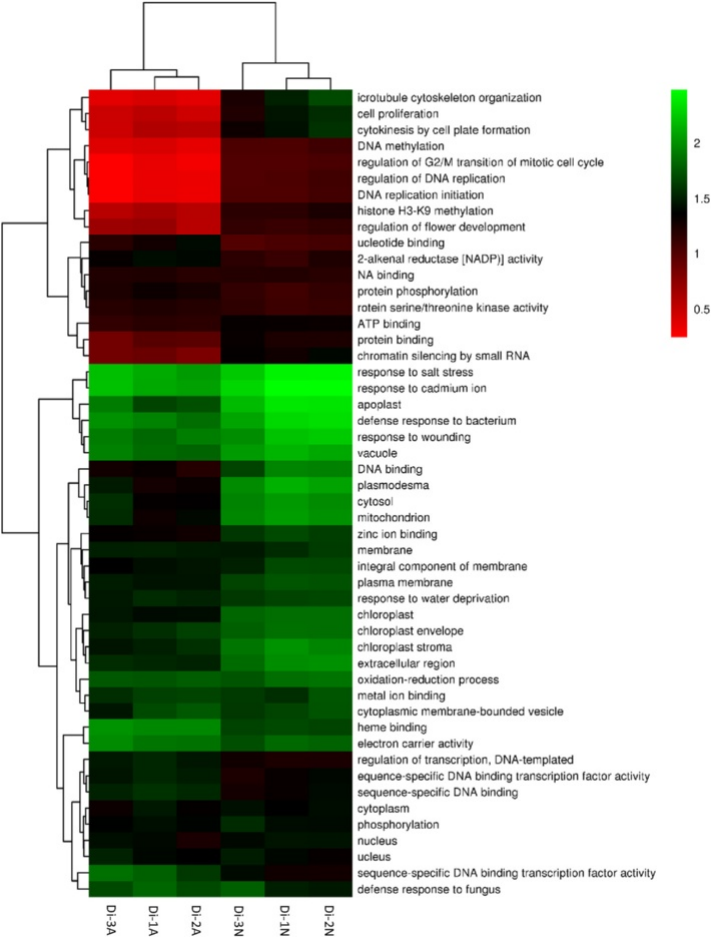
Top 50 different GO terms enriched in normal and abortive seeds. The heat map shows the fold change based on RPKM values of the DEGs in these GO terms in normal and aborted seeds. A q value cutoff of 0.05 calculated by the RPKM of all genes in corresponding GO terms was used to select enriched GO terms

In total, 2770 DEGs were discovered between normal and abortive seeds. Among them, 978 genes were up-regulated and 1792 genes were down-regulated (Fig. 5, Additional file 5). 2631 DEGs were annotated by Nr, Swiss-Prot or genomic data of other species. Top 30 down-regulated and up-regulated genes are shown in Tables 4 and 5, respectively. A total of 1630 genes were annotated by GO. Compared to the unigene library, significantly enriched GO terms were found, such as “protein kinase binding”, “indole-3-acetic acid amido synthetase activity” and “peroxidase activity” in the “molecular function” category. “Cytokinesis by cell plate formation”, “regulation of DNA replication” and “cell proliferation” were found in the “biological process” category. And “nucleosome”, “chromocenter” and “microtubule associated complex” were found in the “cellular component” category. (Fig. 6). The top 50 GO terms for DEGs were shown in Fig. 7.

**Table 4:** Top 30 down-regulated DEGsR

ID	FDR	log_2_FC	Species	Annotation
c21628.graph_c1	2.87E-08	-11.11	*Capsella rubella*	ECAGL3 - ECA1 gametogenesis related family protein precursor
c38878.graph_c0	1.56E-41	-10.57	*Theobroma cacao*	ABI3, putative isoform 1
c34912.graph_c0	9.52E-41	-10.48	*Populus trichocarpa*	GDSL-like Lipase/Acylhydrolase superfamily protein
c20515.graph_c0	5.74E-7	-10.47	*Populus trichocarpa*	bifunctional inhibitor/lipid-transfer protein/seed storage 2S albumin superfamily protein
c32703.graph_c0	2.42E-15	-10.35	*Vitis vinifera*	Lipid transfer protein
c20459.graph_c0	1.33E-41	-10.34	*Ricinus communis*	GDSL-like Lipase/Acylhydrolase superfamily protein
c16559.graph_c0	9.77E-16	-10.31	*Theobroma cacao*	Nuclear factor Y, subunit C2
c42905.graph_c0	8.30E-09	-10.26	*Vitis vinifera*	Late embryogenesis abundant protein-related/LEA protein-related
c17720.graph_c0	1.47E-31	-10.23	*Ricinus communis*	Uncharacterized protein
c28950.graph_c1	1.02E-15	-10.13	*Glycine max*	Bifunctional inhibitor/lipid-transfer protein/seed storage 2S albumin superfamily protein
c32557.graph_c0	3.54E-17	-10.13	*Ricinus communis*	pfkB-like carbohydrate kinase family protein
c17814.graph_c0	2.49E-08	-10.10	*Vitis vinifera*	PEBP (phosphatidylethanolamine-binding protein) family protein
c37825.graph_c0	2.46E-34	-10.10	*Vitis vinifera*	PA-domain containing subtilase family protein
c37890.graph_c0	3.95E-09	-10.05	*Glycine max*	Early nodulin-like protein 9
c40930.graph_c0	4.57E-28	-10.04	*Vitis vinifera*	Core-2/I-branching
				Beta-1,6-N-acetylglucosaminyltransferase family protein
c40314.graph_c0	5.25E-27	-9.99	*Vitis vinifera*	Basic helix-loop-helix (bHLH) DNA-binding superfamily protein
c26337.graph_c0	1.44E-20	-9.95	*Arabidopsis thaliana*	2S sulfur-rich seed storage protein
c17716.graph_c0	2.29E-28	-9.94	*Vitis vinifera*	Uncharacterized protein
c39633.graph_c1	2.40E-11	-9.92	*Sesamum indicum*	RmlC-like cupins superfamily protein
c46499.graph_c1	2.81E-42	-9.87	*Petunia integrifolia subsp. inflata*	RmlC-like cupins superfamily protein
c17661.graph_c0	3.02E-10	-9.83	*Solanum tuberosum*	Aluminium induced protein with YGL and LRDR motifs
c17693.graph_c0	3.89E-23	-9.83	*Magnolia salicifolia*	RmlC-like cupins superfamily protein
c37540.graph_c1	1.61E-10	-9.81	*Setaria italica*	PREDICTED: ZF-HD homeobox protein
c46966.graph_c0	7.42E-45	-9.80	*Prunus persica*	Aquaporin-like superfamily protein
c47330.graph_c0	1.15E-10	-9.80	*Vitis vinifera*	uncharacterized protein
c42176.graph_c0	1.20E-34	-9.77	*Vitis vinifera*	PREDICTED: hydroxycinnamoyl-Coenzyme A shikimate/quinate hydroxycinnamoyltransferase
c40562.graph_c0	6.40E-09	-9.75	*Fragaria vesca subsp. vesca*	Uncharacterized protein
c18277.graph_c0	1.16E-11	-9.69	*Solanum lycopersicum*	Homolog of Medicago truncatula MTN3
c32587.graph_c0	2.25E-37	-9.65	*Populus trichocarpa*	Uncharacterized protein
c17717.graph_c0	1.14E-17	-9.64	*Arabidopsis thaliana*	Seed storage albumin 5

**Table 5:** Top 30 up-regulated DEGs

ID	FDR	log_2_FC	Species	Annotation
c29359.graph_c0	0.003579	8.84	*Vitis vinifera*	FAD-binding Berberine family protein
c40611.graph_c1	0.009777	8.36	*Fragaria vesca subsp. vesca*	PREDICTED: aldehyde dehydrogenase family 2 member B7, mitochondrial-like
c15946.graph_c0	0.000386	7.82	*Fagus crenata*	Transcription factor MYB251
c40565.graph_c0	8.72E-13	7.81	*Solanum lycopersicum*	Soybean gene regulated by cold-2
c31002.graph_c0	0.002082	7.78	*Vitis vinifera*	Heavy metal transport/detoxification superfamily protein
c37038.graph_c1	3.80E-20	7.77	*Ricinus communis*	Auxin-responsive GH3 family protein
c28235.graph_c0	0.001514	7.00	*Diospyros kaki*	Putative MYB transcription factor
c37059.graph_c0	0.001769	6.80	*Populus trichocarpa*	PLANT CADMIUM RESISTANCE 2
c36067.graph_c0	3.16E-20	6.74	*Guillardia theta CCMP2712*	Hypothetical protein GUITHDRAFT_76875, partial
c15251.graph_c0	1.51E-08	6.69	*Vitis vinifera*	Basic helix-loop-helix (bHLH) DNA-binding family protein
c49458.graph_c0	0.001387	6.65	*Theobroma cacao*	Uncharacterized protein
c17425.graph_c0	1.54E-09	6.59	*Solanum lycopersicum*	PREDICTED: CASP-like protein
c39858.graph_c0	2.63E-15	6.52	*Vitis vinifera*	myb domain protein
c35722.graph_c0	0.004717	6.46	*Vitis vinifera*	Laccase-14
c21576.graph_c0	0.00219	6.45	*Vitis vinifera*	Hexose transporter
c48162.graph_c0	1.82E-05	6.42	*Vitis vinifera*	Respiratory burst oxidase protein F
c35307.graph_c0	0.009666	6.40	*Vitis cinerea var. helleri x Vitis riparia*	Tumor-related protein
c15785.graph_c0	0.000308	6.35	*Vitis vinifera*	myb domain protein
c33981.graph_c0	1.23E-05	6.26	*Vitis vinifera*	Nitrate transmembrane transporters
c21046.graph_c0	6.81E-06	6.24	*Vitis vinifera*	Allergen-related protein
c39360.graph_c1	5.14E-14	6.17	*Vitis vinifera*	Squamosa promoter-binding-like protein 8
c17796.graph_c0	3.31E-08	6.16	*Populus trichocarpa*	Uncharacterized protein
c8841.graph_c0	3.04E-07	6.12	*Vitis vinifera*	PREDICTED: protein MKS1-like
c36602.graph_c0	8.46E-09	6.11	*Vitis vinifera*	Lateral organ boundaries (LOB) domain family protein
c45306.graph_c0	2.67E-13	6.09	*Vitis vinifera*	Seven transmembrane MLO family protein
c32231.graph_c0	0.000386	6.01	*Prunus persica*	Uncharacterized protein
c38398.graph_c1	7.24E-20	5.99	*Populus trichocarpa*	Putative membrane lipoprotein
c19374.graph_c0	3.32E-07	5.99	*Theobroma cacao*	myb domain protein
c46658.graph_c0	2.34E-26	5.95	*Theobroma cacao*	Cytokinin oxidase 5
c52887.graph_c0	6.95E-07	5.93	*Vitis vinifera*	FAD-dependent oxidoreductase family protein

**Fig. 8:**
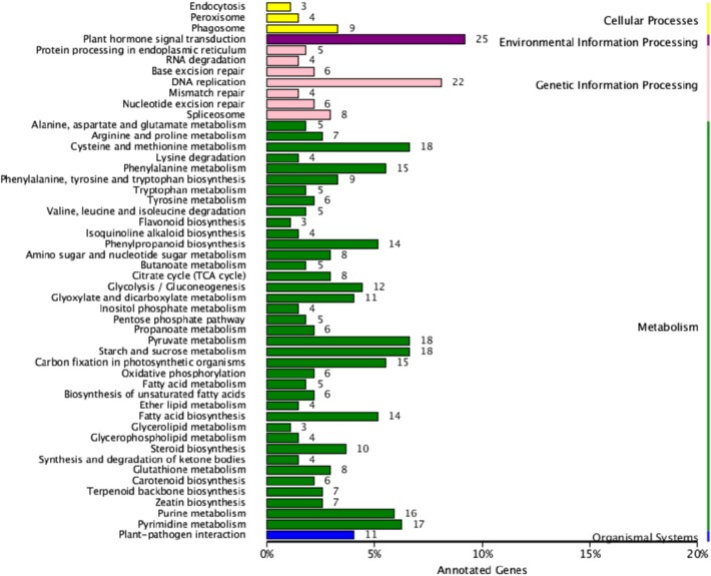
Top 50 enriched KEGG pathways between normal and abortive seeds. Number at the right of each column indicates the number of unigenes included in corresponding pathway. The pathways were divided into five categories: “cellular processes”, “environmental information processing”, “genetic information processing”, “metabolism” and “organismal systems”. A q value cutoff of 0.05 calculated by the RPKM of all genes in corresponding KEGG pathways was used to select enriched KEGG pathways

Then DEGs were aligned to the KEGG database and assigned to 79 pathways. Among them, a large number of genes were involved in the pathways related to metabolism, such as “starch and sucrose metabolism”, “cysteine and methionine metabolism”, “pyruvate metabolism”, “pyrimidine metabolism”, “purine metabolism” and “phenylalanine metabolism”. A number of DEGs enriched in the pathways of genetic information processing, such as “ribosome”, “DNA replication” and “spliceosome” were involved. Another large group of DEGs were enriched in pathways of biosynthesis, including “phenylpropanoid biosynthesis”, “fatty acid biosynthesis”, “steroid biosynthesis”, “terpenoid backbone biosynthesis” and “zeatin biosynthesis”. These results were consistent with the status of normal and abortive seeds, which are quite different in nutrient accumulation, cell proliferation, tissue development and secondary metabolism. Remarkably, a number of genes were enriched in the pathways of “plant hormone signal transduction”, “plant-pathogen interaction”, “endocytosis” and “phagosome”, which are presumed to play critical roles in seed abortion regulation (Fig. 8).

**Fig. 9:**
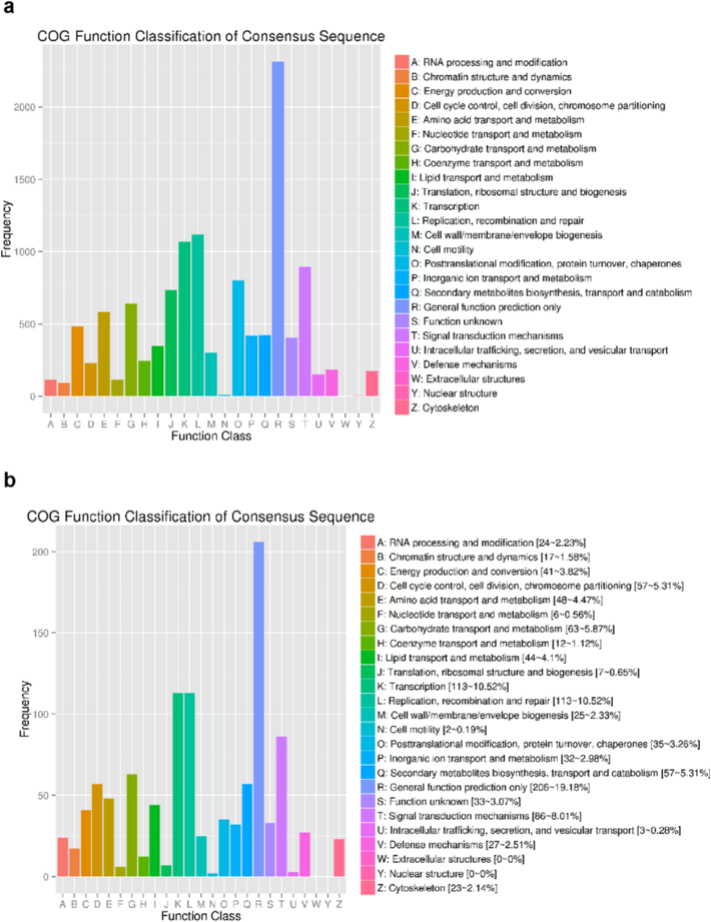
Cluster of orthologous groups (COG) classification. **a** In total, 8,375 of 72,885 unigenes were grouped into 25 COG classifications. **b** 1,074 of the 2,770 DEGs were grouped into 25 COG classifications. DEGs were enriched in the biological processes such as “transcription”, “replication, recombination and repair”, “signal transduction mechanisms” and “carbohydrate transport and metabolism”. Compared to COG analysis of all unigenes, DEGs were significantly enriched in the terms such as “cell cycle control, cell division, chromosome partitioning”, “lipid transport and metabolism” and “secondary metabolites biosynthesis, transport and catabolism”, while less enriched in the terms of “translation, ribosomal structure and biogenesis”, “posttranslational modification, protein turnover, chaperones” and “intracellular trafficking, secretion and vesicular transport”

One thousand seventy-four DEGs were matched in the COG database. Similar to the results of GO and KEGG analysis, COG analysis showed that several DEGs were enriched in the biological processes such as “transcription”, “replication, recombination and repair”, “signal transduction mechanisms” and “carbohydrate transport and metabolism”. On the contrary, the fewest DEGs were enriched in “cell motility”, “intracellular trafficking, secretion, and vesicular transport” and “nucleotide transport and metabolism”. Compared to COG analysis of all unigenes, DEGs were significantly enriched in the terms such as “cell cycle control, cell division, chromosome partitioning”, “lipid transport and metabolism” and “secondary metabolites biosynthesis, transport and catabolism”, while less enriched in the terms of “translation, ribosomal structure and biogenesis”, “posttranslational modification, protein turnover, chaperones” and “intracellular trafficking, secretion and vesicular transport” (Fig. 9).

## Functional analysis of DEGs

### DNA replication and cell proliferation are seriously impaired in abortive seeds

Among the DEGs, all genes encoding DNA polymerase alpha catalytic subunit, DNA replication licensing factor, ATP-dependent DNA helicase, DNA topoisomerase, DNA mismatch repair protein and condensin complex subunit showed dramatically decreased transcript abundance in abortive seeds. Consistently, genes encoding histone, chromatin assembly factor, structural maintenance of chromosomes protein and mini-chromosome maintenance complex-binding protein showed uniformly decreased expression in abortive seeds.

Genes involved in cytokinesis and microtubule cytoskeleton organization, including kinesin-like protein, 125 kDa kinesin-related protein, early nodulin-like protein, 65-kDa microtubule-associated protein, microtubule-associated protein RP/EB family, DNA (cytosine-5)-methyltransferase, high mobility group B protein, MAR-binding filament-like protein, callose synthase, thaumatin-like protein and tubulin showed significantly decreased expression in abortive seeds.

Cell cycle was observed to be disturbed in abortive seeds for nine genes encoding cyclin, four genes encoding G2/mitotic-specific cyclin, two genes encoding cyclin-dependent kinase and eight genes encoding formin-like protein, which were globally down-regulated to a large extent in abortive seeds.

### Fatty acid, starch and sucrose metabolism are at low levels in abortive seeds

**Fig. 10:**
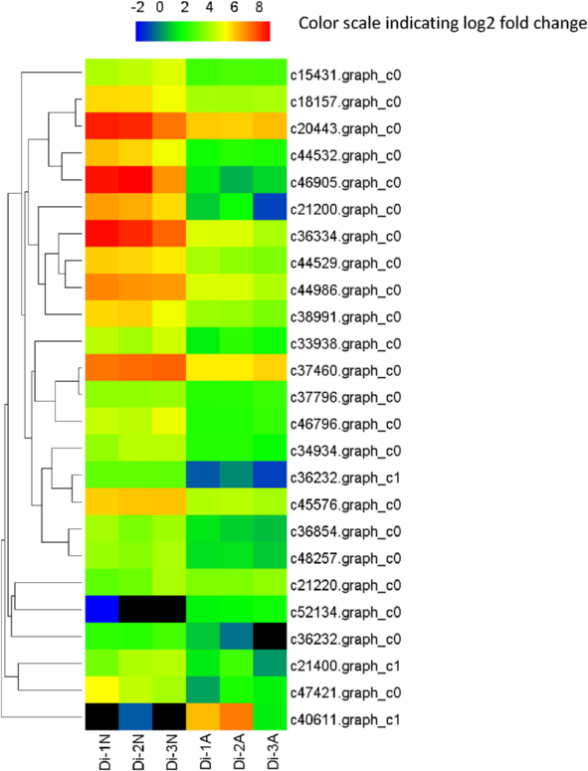
Expression patterns of genes involved in fatty metabolism. The codes of transcripts are indicated at the side of each step. The sample names are showed at the bottom. Black blocks represent that the expression is undetected

Fatty acid content is significantly different between normal and abortive seeds (unpublished data). The high content of fatty acid in normal *Davidia* seed might explain how it survived the Tertiary. Genes involved in fatty acid biosynthesis, including acetyl-coenzyme A carboxylase carboxyl transferase, hydroxyacyl-ACP dehydratase, lipoxygenase, acyl carrier protein, long chain acyl-CoA synthetase and protein ECERIFERUM (which are highly expressed in normal seeds) are uniformly down-regulated in abortive seeds. Furthermore, genes encoding products involved in unsaturated fatty acid biosynthetic process, such as cycloartenol synthase, dihydrolipoyllysine-residue acetyltransferase, peroxygenase, acyl-[acyl-carrier-protein] desaturase, omega-3 fatty acid desaturase and omega-6 fatty acid desaturase are consistently down-regulated in abortive seeds (Fig. 10).

Genes involved in starch biosynthesis and catabolism, such as fructokinase, Alpha-xylosidase, granule-bound starch synthase, catalase and beta-amylase show uniformly decreased transcript abundance, indicating a low starch level in abortive seeds. For sucrose biosynthesis, 3 genes encoding sucrose synthase are dramatically down-regulated in abortive seeds.

Nutrient and ion transport are possibly restricted in abortive seeds. Three genes encoding the bidirectional sugar transporter, three genes encoding the cationic amino acid transporter and three genes encoding the nitrate transporter are significantly down-regulated. Moreover, various genes encoding copper, polyol, boron, zinc, sulfate and potassium transporter, respectively, are also down-regulated at different levels. Moreover, three genes encoding aquaporin are down-regulated to undetected levels in abortive seeds. These results demonstrate that the basic nutrition supply is greatly impaired in abortive seeds.

### Nutrient reservoir and seed development are constrained in abortive seeds

Almost all of the genes encoding seed storage protein, such as globulin, albumin, sulfur-rich seed storage protein and legumin, were found to be down-regulated at the largest degree among DEGs. A number of key regulators of embryo development, such as genes encoding B3 domain-containing protein, ZF-HD homeobox protein, LOB domain-containing protein and zinc finger CCCH domain-containing protein were dramatically down-regulated in abortive seeds. The significantly low expressions of these genes confirmed the developmental defects in abortive seeds.

Notably, two genes encoding protein TRANSPARENT TESTA 12, which is essential for cell elongation in the integument, showed significantly decreased expression. On the other hand, six genes encoding receptor-like protein kinase HAIKU2 (with an exception), which control endosperm growth and modulate integument cell elongation, showed increased expression.

### Difference of plant hormone signal transduction between normal and abortive seeds

Seven genes encoding indole-3-acetic acid-amido synthetase were found in DEGs, five of them showed decreased expression and two of them showed increased expression. Seven genes encoding auxin response factor were found; five of them were down-regulated and two of them were up-regulated. Three genes encoding auxin-responsive protein were found; two of them showed decreased expression. Two genes encoding auxin efflux carrier component were down-regulated. Two genes encoding auxin-induced in root cultures protein were up-regulated.

Three genes encoding gibberellin receptor were up-regulated. Three genes encoding gibberellin 2-beta-dioxygenase were up-regulated while a gene encoding gibberellin 3-beta-dioxygenase was down-regulated. Two genes encoding gibberellin 20 oxidase were undetected in abortive seeds. A number of genes response to gibberellin, such as two genes encoding monogalactosyldiacylglycerol synthase, a gene encoding transcription factor HB29, two gene encoding transcription factor RAX2 and two genes encoding transcription factor TCP15 showed increased expression in abortive seeds.

Five genes encoding cytokinin dehydrogenase/oxidase were found among DEGs; three of them showed increased expression and two of them showed decreased expression. Nine genes encoding ethylene-responsive transcription factor were found, and eight of them showed increased expression with an exception. A gene encoding abscisic acid receptor and two genes encoding abscisic stress-ripening protein showed increased expression.

### Genes involved in response to stress and reactive oxygen species scavenging

Genes response to biotic and abiotic stress included seven genes encoding protein phosphatase 2C, sixteen genes encoding WRKY transcription factor, three genes encoding sugar transport protein, seven genes encoding MYB transcription factor (with an exception), seven genes encoding LRR receptor-like serine/threonine-protein kinase, six genes encoding zinc finger protein ZAT and four genes encoding heavy metal-associated isoprenylated plant protein, showing increased transcript abundance in abortive seeds. These results indicated that abortive seeds were under adversity stress. These up-regulated genes, including various transcription factors and protein kinases, might have initiated corresponding pathways to restrain the growth of the seeds.

Biotic and abiotic stress often induced a high content of reactive oxygen species (ROS) in plants. Genes encoding reactive oxygen species scavengers included a gene encoding cationic peroxidase, four genes encoding respiratory burst oxidase homolog protein and two genes encoding reticuline oxidase-like protein, showing increased expression in abortive seeds. Eleven genes encoding peroxidase were found among DEGs; eight of them showed increased expression and three showed decreased expression.

### Calcium may be an important second messenger in abortion regulation

A number of calcium related genes were found in DEGs. Four genes encoding calcium-binding protein CML, two genes encoding calmodulin-like protein, three genes encoding cation/calcium exchanger and a gene encoding autoinhibited calcium ATPase showed increased expression. Two genes encoding CBL-interacting serine/threonine-protein kinase 14 showed increased expression and one gene encoding CBL-interacting serine/threonine-protein kinase 7 showed decreased expression. On the other hand, a gene encoding calcium-dependent protein kinase, a gene encoding calmodulin-binding transcription activator and three genes encoding calreticulin showed decreased expression. These results implied that calcium played an important role in abortion regulation.

### Cell apoptosis and programmed cell death in abortive seeds

A number of genes involved in cell apoptosis, such as a gene encoding BAG family molecular chaperone regulator and nine genes encoding CASP-like protein (with an exception) showed increased expression in abortive seeds. Ten genes encoding F-box protein that might have participated into the apoptosis process were also found. Six showed increased expression and four showed decreased expression.

Genes involved in programmed cell death, including six genes encoding aspartic proteinase, four genes encoding cysteine-rich receptor-like protein kinase, a gene encoding leucine-rich repeat receptor-like protein kinase PXL2 and two genes encoding NAC transcription factor, were found to be significantly up-regulated in abortive seeds. Remarkably, a gene encoding cysteine proteinase showed decreased expression, while three genes encoding cysteine proteinase inhibitor showed dramatically decreased expression, indicating subtle mechanisms in cysteine proteinase activity regulation.

### Lignin biosynthesis and secondary cell wall biogenesis

**Fig. 11:**
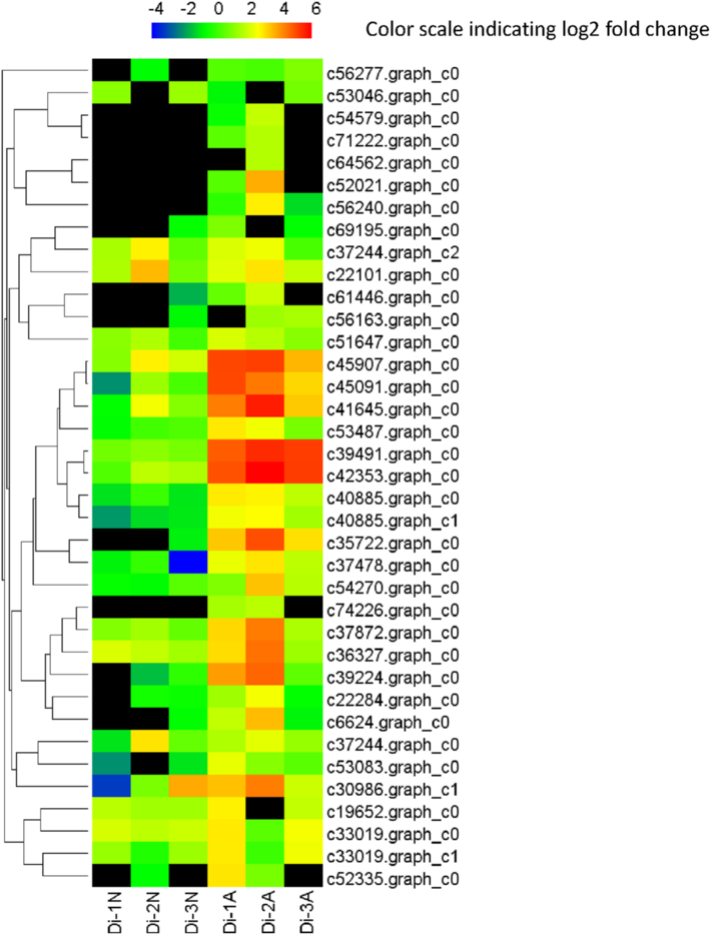
Expression pattern of laccase genes in normal and abortive seeds. The codes of transcripts are indicated at the side of each step. The sample names are showed at the bottom. Black blocks represent that the expression is undetected

Almost all DEGs involved in lignin biosynthesis showed significantly increased expression, including seven genes encoding laccase, a gene encoding caffeoyl-CoA O-methyltransferase, a gene encoding cinnamyl alcohol dehydrogenase, a gene encoding shikimate O-hydroxycinnamoyltransferase and a gene encoding COBRA-like protein. On the other hand, various genes involved in secondary cell wall biogenesis showed uniformly increased expression, including five genes encoding cellulose synthase A catalytic subunit, a gene encoding secondary cell wall-related glycosyltransferase, a gene encoding protein IRX15-like, a gene encoding UDP-glucuronate:xylan alpha-glucuronosyltransferase and two genes encoding NAC domain-containing protein. Some previously described genes, such as genes encoding COBRA-like protein, MYB transcription factor and laccase, were also involved in secondary cell wall biogenesis. Laccase family is also involved in the oxidation-reduction process. We compared the expression of all identified *Davidia laccase* genes between normal and abortive seeds, and the results demonstrated that they were significantly up-regulated in abortive seeds (Fig. 11). Moreover, we found eight genes encoding wall-associated receptor kinase were uniformly up-regulated in abortive seeds. These results indicated that lignin accumulation and cell wall organization were changed in abortive seeds, critically affecting the regulation of seed development.

### Validation of differentially expressed genes by qPCR

**Fig. 12:**
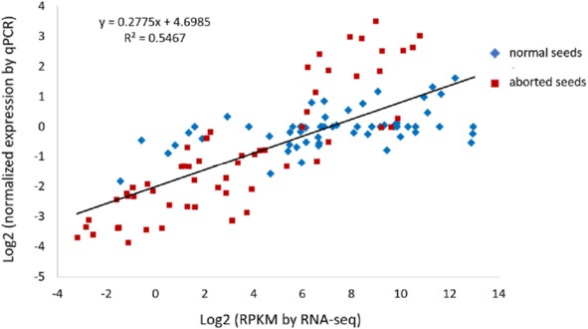
Correlation of gene expression results. The x-axis represents the value of Log2 RPKM and the y-axis represents the value of Log2 normalized expression level. Violet diamonds represent normal seeds. Red squares represent aborted seeds. R^2^ value represent the correlation between RNA-seq and qPCR results

To confirm the accuracy and reproducibility of the RNA-Seq results, 19 genes (detailed information is shown in Additional file 3: Table S1) with distinct expression profiles in normal and abortive seeds were chosen for qPCR validation. The expression levels of selected genes in normal and aborted seeds were detected by qPCR (Additional file 6). Correlation between RNA-Seq results (RPKM) and qPCR results (2^-ΔΔCT^) was calculated using the log2 fold change measurements to generate the scatterplots. The results showed that the qPCR results had significant similarity (*r*^2^ = 0.55) with the RNA-Seq data (Fig. 12).

## Discussion

### *Davidia* transcriptome

As an ancient relic species, *Davidia* has unique features in its fruits, seeds and bracts. The endosperm of normal *Davdia* seeds has abundant fatty acid and storage protein, which makes it nutritious and cold-resistant. During fruit development, lignin rapidly accumulates in the endocarp after approximately 20 days. This process makes the endocarp extremely hard and the space between the endocarp and seeds nearly contiguous (Fig. 2). These properties were presumed to be important reasons why *Davida* was able to survive the Quaternary Period [1]. *Davidia* transcriptome analysis showed that corresponding genes involved in fatty acid metabolism and lignin biosynthesis was highly expressed. High activity of secondary metabolites biosynthesis such as phenylpropanoid, flavonoid, terpene and steroid were indicated from the expression levels of related genes, implying that diverse secondary metabolites exist in *Davidia*. Compared to the genomic data of *Arabidopsis thaliana*, *Vitis vinifera*, *Theobroma cacao*, *Populus trichocarpa*, *Eucalyptus grandis* and *Amborella trichopoda*, *Davidia* sequences showed relatively low similarity to them (Additional file 4: Table S2), indicating its unique position in the evolution of angiosperm. Only 45.6 % transcripts were annotated, which was relatively low, suggesting that plenty of novel genes (which should include genes closely related to the unique phynotype of the fruit and seed of *Davidia*) were discovered from our data. In addition, approximately 0.2 % sequences were homologous to algae, bacteria, fungi and yeast, implying genetic integration events occurred, or multiple endogenous microorganism existed in *Davidia*.

### Possible inducements of seeds abortion in *Davidia*

The serious seed abortion in *Davida* has been reconfirmed through our investigations. The breeding system of dove tree is an outcrossing type and partially self-compatible. It produces excessive flowers and pollens in the reproduction period, and the pollination rate is very high [25]. No abnormal development has been observed in gametogenesis or female and male gamete development [26]. Therefore, inbreeding depression is considered to be an important reason for abortion. The identified down-regulated genes, which are involved in energy and metabolic pathways, confirm the low vigor and rivalry power of abortive seeds, which might due to the genetic load caused by inbreeding.

The alteration of phytohormone levels was a significant sign in abortive seeds. From our results, major indole-3-acetic acid-amido synthetase genes and auxin response factor genes were significantly down-regulated. On the contrary, ethylene-responsive transcription factor genes and gibberellin receptor genes were globally up-regulated. These results implied that auxin levels were decreased, while gibberellin, ethylene and ABA levels were increased in abortive seeds. Gibberellins are essential regulators in plant development. Ectopic expression of a pea GA 2-oxidase2 cDNA induced gibberellin-deficiency and caused seed abortion in Arabidopsis [27]. It was also reported that high levels of gibberellin could cause seed abortion in grape [28]. Similarly, high levels of ethylene could induce kernel abortion and suppression of grain maturation in wheat [29]. However, the role of gibberellin and ethylene in seed formation and germination has remained controversial due to different results observed in different plant species [30]. ABA level was closely related to the response to stress in plants, and the increased ABA levels had been described in aborted seeds of maize and chrysanthemum [23, 31].

### Signal transduction messagers involved in seed abortion

It is critical for maternal plants to recognize “bad offspring” and selectively restrain their growth. How the messages are exchanged between seeds and maternal plants remains unclear. According to our data, we infer that calcium ion is an important messenger in the abortion pathway. The increased expression of cation/calcium exchanger should change the Ca^2+^ levels in the seeds and regulate the activity of Ca^2+^-dependent protein kinases, subsequently controlling the seeds’ development [32].

Sucrose, which plays a critical role in seed development, is presumed to be another molecular messenger in abortion regulation. Suppression of *sucrose synthase* gene expression in cotton inhibits endosperm and embryo development and blocks the formation of adjacent seed integument transfer cells [33]. Overexpression of a potato sucrose synthase gene in cotton improved early seed development and reduced seed abortion [34]. We identified three genes encoding sucrose synthase that showed more than a 100-fold decreased expression in abortive seeds, demonstrating that the deficiency of sucrose synthase activity was critical for seed abortion in *Davidia*.

### Programmed cell death in abortive seeds

Programmed cell death (PCD) is closely related to the vegetative and reproductive development of a plant [35]. Cysteine and aspartic proteinases are essential protelytic enzymes involved in PCD [36]. We identified five genes encoding aspartic proteinase that were uniformly up-regulated in abortive seeds, demonstrating the regulatory roles of this gene family in abortive seeds. Similar results were reported in comparative proteomic analysis of longan seed abortion, in which three cysteine protease protein were highly accumulated in abortive seeds at 50 d after pollution, suggesting that PCD was a common mechanism of seed abortion in different species [21]. Interestingly, we found one *cysteine proteinase* gene that was down-regulated in abortive seeds while three *cysteine proteinase inhibitor* genes were drastically down-regulated in abortive seeds. This finding indicated that cysteine proteinase activity was subtly regulated by its inhibitor in *Davidia*.

### Lignin biosynthesis and seed integument development

We identified a series of genes related to secondary cell wall biogenesis and lignin biosynthesis that were significantly up-regulated in abortive seeds. *Laccase* genes were the most significant DEGs found to have uniformly improved expression levels in abortive seeds (Fig. 11). Laccase is a multiple function enzyme that can induce the flavonoid oxidation, which is also a resistance mechanism against biotic and abiotic stress [37]. The function of laccase was nonredundant with peroxidase for lignin polymerization [38], and most *peroxidase* genes were also significantly up-regulated in abortive seeds. A gene family encoding cellulose synthase A catalytic subunit, also involved in the lignin biosynthetic pathway, was found uniformly up-regulated in abortive seeds. Notably, some laccase and cellulose synthase were specially expressed in seed integument [37, 39]. Maternal control of integument cell elongation was validated to determine seed size in Arabidopsis [40]. Significantly decreased expression of protein TRANSPARENT TESTA indicated the development of integument was restricted in abortive seeds. Altogether, we assumed the growth of abortion seeds were controlled by maternal plants through the seed integument. The rapid accumulation of lignin or cellulose might have occurred in the seed integument, thus forming a compact and hard structure, which would restrain endosperm development.

### Candidate regulators in seed abortion

For transcription factor, most stress-responsive transcription factors, such as AP2, MYB and WRKY transcription factor showed uniformly increased expression. Most development-related transcription factors, such as B3 domain-containing transcription factor, showed decreased expression in abortive seeds.

MYB domain protein was reported to act as a key determinant for proanthocyanidin accumulation [41]. Related genes, including three genes encoding anthocyanin regulatory C1 protein also showed increased expression. Proanthocyanidin accumulation was involved in seed integument development of Arabidopsis [42]. Some MYB transcription factors were also involved in lignification and secondary cell wall formation [43, 44].

Two *Arabidopsis* genes, *MINISEED3* (*MINI3*) and *HAIKU2* (*IKU2*), are proven regulators of seed size [45]. *MINISEED 3* encodes Arabidopsis AtWRKY10, and a *wrky10* mutant produces significantly smaller seeds. *HAIKU2* encodes a protein kinase, and the *haiku* mutant produces seeds of reduced size, which results from impaired communication between the endosperm and maternal seed integument. Interestingly, among almost all *WRKY* genes and the six *HAIKU2* genes in our DEG data, most genes showed increased expression. Only one *HAIKU2* gene showed decreased expression. These findings implied different regulatory mechanisms in *Davidia*.

It is notable that most wall-associated receptor kinases, which are required for cell expansion and disease resistance [46], show uniformly increased expression in abortive seeds. Whether this gene family is involved in the signal transduction of abortion needs further investigation.

The genetic transformation system of *Davidia* is not available; therefore, further study on the function of the candidate gene, especially up-regulated transcription factors and gene families, should be performed in other species such as Arabidopsis. On the other hand, our data indicates limited nutrient and phytohormone regulation is essential for abortion. Therefore, exogenous nutrients and exogenous hormone imposing might be effective methods to alleviate abortion. If seed abortion in *Davidia* can be alleviated, it will bring great advantages for propagation and conservation of the tree.

## Conclusion

*De novo* transcriptome sequencing of *Davidia involucrata* Baill. was performed in the present study using Illumina paired-end sequencing technology. In total, 72,885 unigenes from the fruits and seeds of *Davidia* were isolated. Focus on the regulatory mechanism of serious seed abortion in *Davidia*, the differentially expressed genes between normal and abortive seeds, were analyzed. We proposed that genetic load, resource limitation and phytohormone regulation were critical determinants for *Davidia* seed abortion. According to gene expression profiles, biological processes such as response to stress, starch and sucrose metabolism, PCD, secondary cell wall biogenesis and lignin biosynthesis were identified to be critical for abortion regulation. We assumed that maternally controlled development of integument was a critical process for abortion regulation. Calcium and sucrose were proposed to be important messengers in the abortion pathway. MYB and WRKY transcription factors, receptor kinase and laccase were identified as candidate regulators in seed abortion. The genomic data of *Davidia* will facilitate the further research on such endangered and low-fecundity species, and provide theoretical basis for protecting and utilizing these precious resources.

## Methods

### Plant materials

The fruits and seeds of *Davidia* were collected from three individual flowering trees of the naturally distributed *Davidia* population at Badagong Moutain Natural Reserve, Sangzhi County, Hunan Province (110°5’30”E, 28°46’60”N, 1383 m altitude). To eliminate genetic variance, we selected three trees that were grafted at the same time in 1983. The scions used for grafting were collected from the identical plant, known as the “King of Dove Trees”, the oldest dove tree in China (approximate age is 400 years). The fruits were collected on July 14, 2014, approximately 1 month after the bracts abscission, which represented the rough age of the seeds, or 60 to 90 days. The seeds were obtained by immediately dissecting the fruits after collection. Abortion ratio was calculated by N_A_/N_T_. (N_A_, number of abortive seeds in a fruit; N_T_, number of total seeds in a fruit). The normal seeds, abortive seeds and other parts of the fruit were separated and quickly frozen in liquid nitrogen and stored at −80 °C. Another group of the collected seeds were immediately fixed in formalin-aceto-alcohol (FAA) for microscopic observation. Three fruits of each tree were collected for seed sample preparation. Total normal seeds (3–5 grains) and total abortive seeds (15–20 grains) from the identical fruits were mixed and prepared as individual samples, respectively. The seed samples were named Di-1 N (mixed normal seeds from fruits of tree 1), Di-1A (mixed abortive seeds from the same fruits of tree 1), Di-2 N (mixed normal seeds from fruits of tree 2), Di-2A (mixed abortive seeds from the same fruits of tree 2), Di-3 N (mixed normal seeds from fruits of tree 3), and Di-3A (mixed abortive seeds from the same fruits of tree 3). All collected fruit samples (with seeds removed) were mixed and named as Di-F.

### Microscopic observation

The normal and abortive seeds were fixed in FAA (50 % alcohol: acetic acid: formaldehyde solution = 89: 6: 5) immediately after dissection and stored at room temperature. Samples were washed in 50 % alcohol, dehydrated using an ethyl alcohol series, cleared in xylene and embedded in paraffin wax. The specimens were sectioned to a thickness of 8 μm. Sections were stained with hematoxylin, examined and photographed using an OLYMPUS BX-51 microscope.

### RNA extraction,quantification and qualification

Total RNA was extracted using E.Z.N.A. Plant RNA Kit (Omega, R6827-01). RNA degradation and contamination was monitored on 1 % agarose gels. RNA purity was checked using the NanoPhotometer® spectrophotometer (IMPLEN, CA, USA). RNA concentration was measured using Qubit® RNA Assay Kit in Qubit®2.0 Flurometer (Life Technologies, CA, USA). RNA integrity was assessed using the RNA Nano 6000 Assay Kit of the Agilent Bioanalyzer 2100 system (Agilent Technologies, CA, USA).

### Library construction and RNA-Seq

Construction of the library and RNA-Seq was performed by Biomarker Biotechnology Corporation (Beijing, China). RNA of the fruits, normal seeds and abortive seeds were combined in equal quantity to construct a large pool. Sequencing libraries were generated using NEBNext®Ultra™ RNA Library Prep Kit for Illumina® (NEB, USA) following the manufacturer’s recommendations. Briefly, mRNA was purified from total RNA using poly-T oligo-attached magnetic beads. Fragmentation was carried out using divalent cations under elevated temperature in NEBNext First Strand Synthesis Reaction Buffer (5X). First strand cDNA was synthesized using random hexamer primer and M-MuLV Reverse Transcriptase (RNase H). Second strand cDNA synthesis was subsequently performed using DNA Polymerase I and RNase H. Remaining overhangs were converted into blunt ends via exonuclease/polymerase activities. After adenylation of 3’ ends of DNA fragments, NEBNext Adaptor with hairpin loop structure were ligated to prepare for hybridization. To select cDNA fragments at a preferential length of 150 ~ 200 bp, library fragments were purified with AMPure XP system (Beckman Coulter, Beverly, USA). Then 3 μl USER Enzyme (NEB, USA) was used with size-selected, adaptor-ligated cDNA at 37 °C for 15 min followed by 5 min at 95 °C before PCR. Then PCR was performed with Phusion High-Fidelity DNA polymerase, Universal PCR primers and Index (X) Primer. At last, PCR products were purified (AMPure XP system) and library quality was assessed on the Agilent Bioanalyzer 2100 system. The cDNA library was sequenced on Illumina HiSeq™ 2500 using paired-end technology in a single run.

### Sequence analysis and *De novo* assembly

Clean data was obtained by removing reads containing adapter, reads containing ploy-N and low quality reads from raw data. The clean reads were assembled into contigs using the Trinity method, which recovers more full-length transcripts across a broad range of expression levels, with sensitivity similar to methods that rely on genome alignments [47]. We used the Trinity method with an optimized k-mer length of 25 for *de novo* assembly. Subsequently, the contigs were linked into transcripts according to the paired-end information of the sequences. Transcripts were then clustered based on nucleotide sequence identity. The longest transcripts in the cluster units were regarded as unigenes to eliminate redundant sequences, and then the unigenes were combined to produce the final assembly used for annotation.

### Gene functional annotation

All the assembled unigenes were searched against the Nr (NCBI non-redundant protein sequences) database to identify the putative mRNA functions using the BLAST algorithm [48] with an E-value cut-off of 10^−5^. The BLAST algorithm was also used to align unique sequences to the Nt (NCBI non-redundant nucleotide sequences) and Swiss-Prot (a manually annotated and reviewed protein sequence database). Additionally, to improve the accuracy of the annotation, the assembled unigenes were aligned against the available genomic data of several species, including *Arabidopsis thaliana* (http://www.arabidopsis.org/), *Populus trichocarpa*, *Vitis vinifera*, *Theobroma cacao*, *Eucalyptus grandis* and *Amborella trichopoda* (http://phytozome.jgi.doe.gov/pz/portal.html).

GO (Gene Ontology) terms were extracted from the best hits obtained from the BLASTx against the Nr (non-redundant protein database) using the Blast2GO program [49]. COG (Clusters of Orthologous Groups of proteins) and KO (KEGG Ortholog database [50]) (with E-value cut-off of 10^−5^) analysis was conducted to predict possible functional classifications and molecular pathways.

### Differential gene expression analysis

All reads from three normal seed samples and three abortive seed samples were mapped onto the nonredundant reference transcriptome by Tophat Bowtie software [51] to quantify the abundance of transcripts. Uni-transcript abundance differences between the samples were calculated based on the ratio of the RPKM values [52], and the false discovery rate (FDR). Differential expression analysis of normal and abortive seeds was performed using the DESeq R package (1.10.1). DESeq provided statistical routines for determining differential expression in digital gene expression data using a model based on the negative binomial distribution. The resulting P values were adjusted using Benjamini and Hochberg’s approach for controlling the false discovery rate. Genes with an adjusted *P*-value <0.05 found by DESeq were assigned as differentially expressed. Uni-transcripts with an absolute value of log2 ratio ≥2 and an FDR significance score <0.001 was used for subsequent analysis. The identified DEGs were performed with GO, KEGG and COG analysis using the method described in the “Gene functional annotation” section. GO terms and KEGG pathways with a corrected *P*-value <0.05 (calculated by RPKM of genes involved in) were identified as differentially expressed.

### qPCR analysis

The extracted RNA of seed samples were converted into cDNA using PrimeScript^TM^ One Step RT-PCR Kit Ver. 2 (Takara, Japan). Then the cDNA were 10 × diluted and used as templates for qPCR. qPCR reaction was performed using 2 × SYBR Green qPCR Master Mix (Biotool, USA) on ABI StepOne^TM^. Two independent biological replicates of each sample and three technical replicates of each biological replicate were used for qPCR analysis. A *Davidia* gene, *DiActin*, was used as the reference gene for data normalization. Primers used in qPCR are shown in Additional file 7. The relative expression fold of each sample was calculated by its C_T_ value normalized to the C_T_ value of reference gene using the 2^-ΔΔCT^ method described by Livak and Schmittgen, 2001 [53]. The normalized values of relative expression and RPKM values were calculated by log2, respectively, and the values were used to analyze the correlation between qPCR and RNA-seq results.

### Ethics

The authority responsible for the Davidia resources is the Badagong Mountain Nature Reserve Management Division, who provided permission to collect the samples for our scientific research.

### Consent to publish

Not applicable.

## Availability of data and materials

The sequencing raw data was deposited to the NCBI Short Reads Archive (SRA) with the accession number SRP058736. The BioSample accession is SAMN03733273 and the BioProject ID is PRJNA284915. The data was set to be released at 2018-5-24.

## Additional files

Additional file 1: Sequences of all assembled unigenes. (FA 48010 kb) Additional file 2 All annotated unigenes. (XLS 29890 kb) Additional file 3: Table S1. Summary for the annotation of unigenes against database of other species. (PDF 9 kb) Additional file 4: Table S2. Correlation coefficient between RPKM of unigenes of samples. (PDF 93 kb) Additional file 5: DEGs between normal and abortive seeds. (XLS 1148 kb) Additional file 6: Figure S1. Gene expression in normal and aborted seeds detected by qPCR. (PDF 313 kb) Additional file 7: Table S3. Primer sequences used in qPCR. (PDF 34 kb)

## Abbreviations

*Davidia*: *Davidia involucrata* Baill.
DEG: differentially expressed gene
PCD: programmed cell death
