# Supplementary material for: De novo transcriptome sequencing and gene expression analysis reveal potential mechanisms of seed abortion in dove tree (Davidia involucrata Baill.)
Source: BMC Plant Biol. 2016 Apr 12;16:82. doi: 10.1186/s12870-016-0772-x (PMC4828838; doi:10.1186/s12870-016-0772-x)
Supplement: Additional file 3: Table S1. — Summary for the annotation of unigenes against database of other species. (PDF 9 kb) [file 12870_2016_772_MOESM3_ESM.pdf]

Table S1. Summary for the annotation of unigenes against database of other species

Species	Unigene	Annotation proportion
Arabidopsis thaliana	26,085	35.30%
Vitis vinifera	28,809	38.99%
Theobroma cacao	29,450	39.86%
Populus trichocarpa	28,093	38.02%
Eucalyptus grandis	26,534	35.91%
Amborella trichopoda	25,247	34.17%
