# Supplementary material for: De novo transcriptome sequencing and gene expression analysis reveal potential mechanisms of seed abortion in dove tree (Davidia involucrata Baill.)
Source: BMC Plant Biol. 2016 Apr 12;16:82. doi: 10.1186/s12870-016-0772-x (PMC4828838; doi:10.1186/s12870-016-0772-x)
Supplement: Additional file 4: Table S2. — Correlation coefficient between RPKM of unigenes of samples. (PDF 93 kb) [file 12870_2016_772_MOESM4_ESM.pdf]

Table S2. Correlation coefficient between RPKM of unigenes of samples

Samples	Di-1N	Di-1A	Di-2N	Di-2A	Di-3N	Di-3A
Di-1N	1	0.0187	0.8252	0.0099	0.8298	0.0186
Di-1A	0.0187	1	0.0553	0.9601	0.0807	0.9598
Di-2N	0.8252	0.0553	1	0.0299	0.7831	0.0542
Di-2A	0.0099	0.9601	0.0299	1	0.06	0.9426
Di-3N	0.8298	0.0807	0.7831	0.06	1	0.0915
Di-3A	0.0186	0.9598	0.0542	0.9426	0.0915	1
