# Supplementary material for: De novo transcriptome sequencing and gene expression analysis reveal potential mechanisms of seed abortion in dove tree (Davidia involucrata Baill.)
Source: BMC Plant Biol. 2016 Apr 12;16:82. doi: 10.1186/s12870-016-0772-x (PMC4828838; doi:10.1186/s12870-016-0772-x)
Supplement: Additional file 6: Figure S1. — Gene expression in normal and aborted seeds detected by qPCR. (PDF 313 kb) [file 12870_2016_772_MOESM6_ESM.pdf]

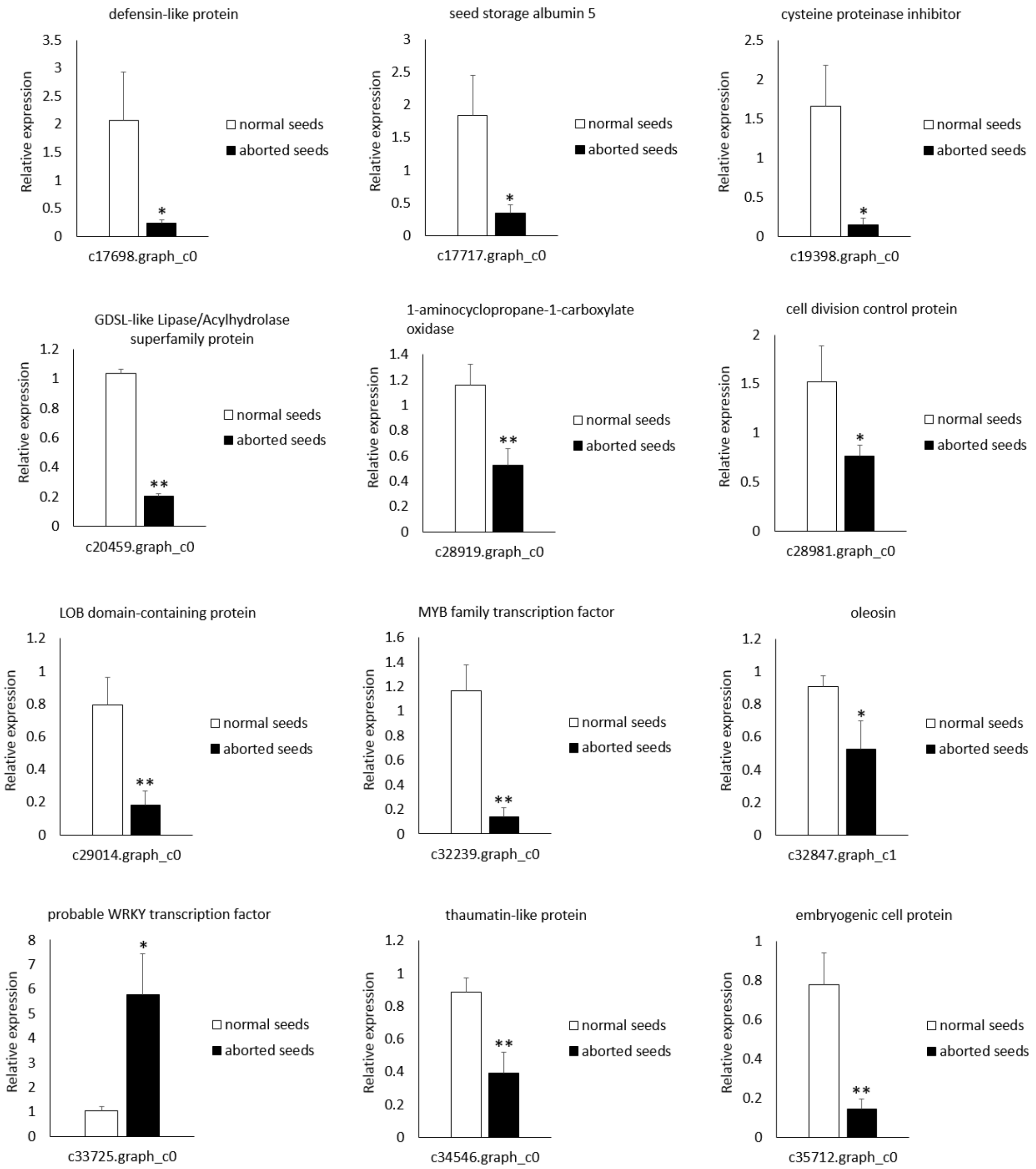
defensin-like protein
c17698.graph_c0
seed storage albumin 5
c17717.graph_c0
cysteine proteinase inhibitor
c19398.graph_c0
GDSL-like Lipase/Acylhydrolase superfamily protein
c20459.graph_c0
1-aminocyclopropane-1-carboxylate oxidase
c28919.graph_c0
cell division control protein
c28981.graph_c0
LOB domain-containing protein
c29014.graph_c0
MYB family transcription factor
c32239.graph_c0
oleosin
c32847.graph_c1
probable WRKY transcription factor
c33725.graph_c0
thaumatin-like protein
c34546.graph_c0
embryogenic cell protein
c35712.graph_c0
Relative expression
normal seeds
aborted seeds

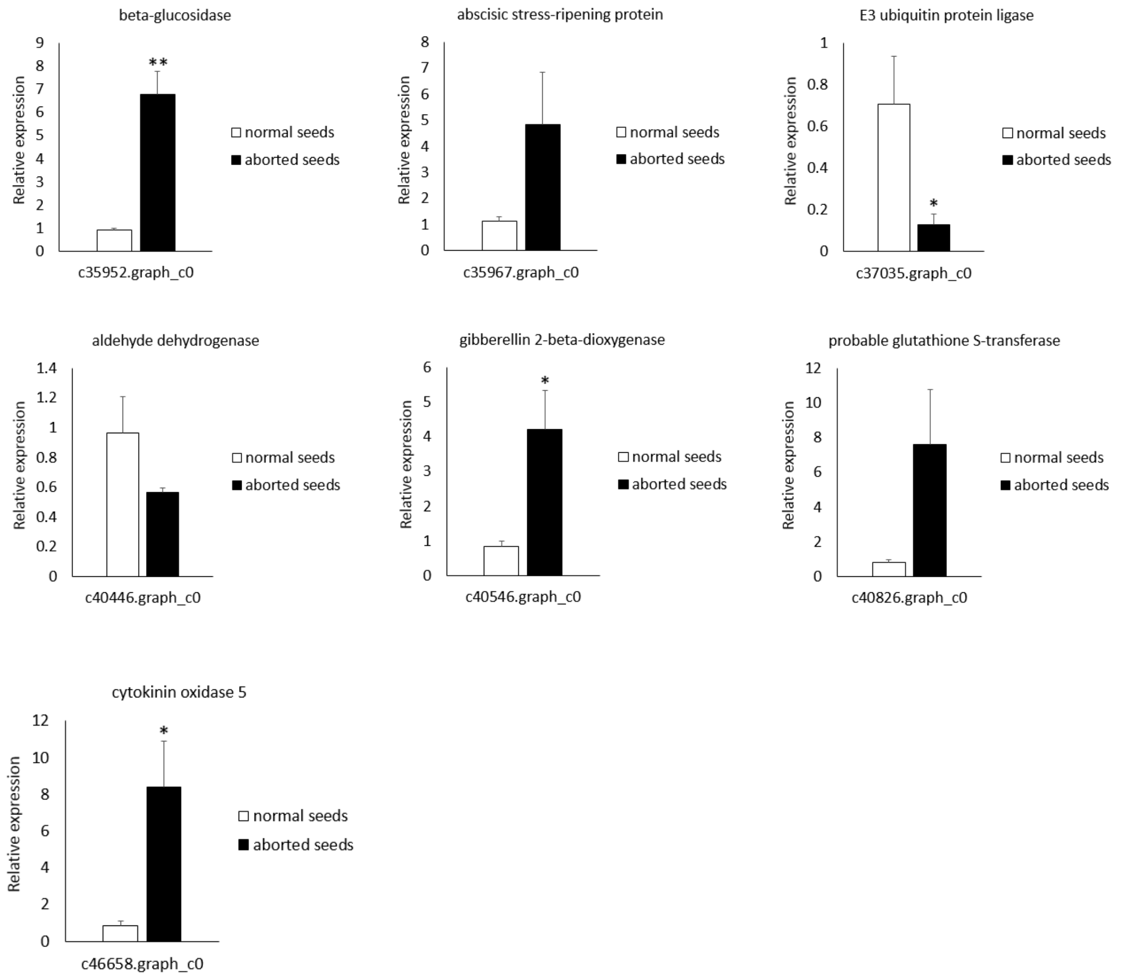

Figure S1. Expression levels of DEGs in normal and aborted seeds detected by qPCR

The results were obtained from three independent seed samples. Error bars are means ±SD of three replicates. The significance between the samples was tested by ANOVA. **Significant at $p < 0.01$ level; *significant at $p < 0.05$ level.
