# Supplementary material for: De novo transcriptome sequencing and gene expression analysis reveal potential mechanisms of seed abortion in dove tree (Davidia involucrata Baill.)
Source: BMC Plant Biol. 2016 Apr 12;16:82. doi: 10.1186/s12870-016-0772-x (PMC4828838; doi:10.1186/s12870-016-0772-x)
Supplement: Additional file 7: Table S3. — Primer sequences used in qPCR. (PDF 34 kb) [file 12870_2016_772_MOESM7_ESM.pdf]

Table S3. Primer sequences used in qPCR

Primer	Sequence	Target	Annotation
17698F	CAATGAGAAGCAAGAAGA	c17698.graph_c0	defensin-like protein
17698R	TTGTCTGTGTGAAAGTTT		
17717F	CAAAGCTCACAGTCGTAG	c17717.graph_c0	seed storage albumin 5
17717R	TTATAGTGGTGCGGTAGG		
19398F	GAAGCATCATAGGAGTCT	c19398.graph_c0	cysteine proteinase inhibitor
19398R	TTGTAACCCATAATTCTACTG		
20459F	CCTTAACATACCTGACATTA	c20459.graph_c0	GDSL-like Lipase/Acylhydrolase superfamily protein
20459R	ACCAACGAGTAGTCTATT		
28919F	AATGGCAAGTATAAGAGTGT	c28919.graph_c0	1-aminocyclopropane-1-carboxylate oxidase
28919R	GGATTGTAGAATGAGGCTAT		
28981F	TCAACTCATTACTCAACT	c28981.graph_c0	cell division control protein
28981R	CCCAACAACCTAAATATG		
29014F	TTCTGGCGAATCATCAAC	c29014.graph_c0	LOB domain-containing protein
29014R	GGCTCATTGTCATTTCCT		
32239F	GAAGTTCAGACTAGGTAG	c32239.graph_c0	MYB family transcription factor
32239R	TTCATCATTCACATCAGA		
32847F	GCGATAACAGTGTTCTTT	c32847.graph_c1	oleosin
32847R	GATCCATGACAGTACAGT		
33725F	CAAGTTCAACGCTTATCG	c33725.graph_c0	probable WRKY transcription factor
33725R	GTAGACTTCTCAATAGGATGT		
34546F	GCAGAAGGTGACCAAATAG	c34546.graph_c0	thaumatin-like protein
34546R	GGCTTACTCTTATGCTTATGAT		
35712F	GTAACGGAAATTCAGACT	c35712.graph_c0	embryogenic cell protein
35712R	AATATCACGATAATAGTAGTAGT		
35952F	TACCTTGAACACCATCTT	c35952.graph_c0	beta-glucosidase
35952R	TGTAACCTTGACTCCATT		
35967F	CACCACCACCTCTTACAC	c35967.graph_c0	abscisic stress-ripening protein
35967R	GTGCTTCTCCTCTTTCTTGT		
37035F	GGCTACCTCAATCAATCTC	c37035.graph_c0	E3 ubiquitin protein ligase
37035R	CCTAAGTCCTCGTTACATAC		
40446F	CATACCTGTTACACTTGAG	c40446.graph_c0	aldehyde dehydrogenase
40446R	GTTCTGTCCACTTGATTG		
40546F	CTAACAGCCAATCCAGAA	c40546.graph_c0	gibberellin 2-beta-dioxygenase
40546R	GAATCTCACAAGCCATCT		
40826F	TACGAATACATTGAACAAG	c40826.graph_c0	probable glutathione S-transferase
40826R	GGACACTCTTATGGATAG		
46658F	CGAATCAACCGCCAATAC	c46658.graph_c0	cytokinin oxidase 5
46658R	CCACATAAGGAAGGTCAGT		
DiActinF	GGTCGTACAACTGGTATT	c45663.graph_c0	actin
DiActinR	GAGCATAGCCTTCATAGAT		
